# Reinvestigation of the relationship between the amplitude of the first heart sound to cardiac dynamics

**DOI:** 10.1002/phy2.53

**Published:** 2013-08-22

**Authors:** Hong Tang, Chengjie Ruan, Tianshuang Qiu, Yongwan Park, Shouzhong Xiao

**Affiliations:** 1 Department of Biomedical Engineering, Dalian University of TechnologyDalian, China; 2 Department of Information and Communication, Yeungnam UniversityDaegu, Korea; 3 College of Bioengineering, Chongqing UniversityChongqing, China

**Keywords:** Amplitude of the first heart sound, exponential model, model fitting, rise rate of left ventricular pressure

## Abstract

The relationships between the amplitude of the first heart sound (S1) and the rising rate of left ventricular pressure (LVP) concluded in previous studies were not consistent. Some researchers believed the relationship was positively linear; others stated the relationship was only positively correlated. To further investigate this relationship, this study simultaneously sampled the external phonocardiogram, electrocardiogram, and intracardiac pressure in the left ventricle in three anesthetized dogs, while invoking wide hemodynamic changes using various doses of epinephrine. The relationship between the maximum amplitude of S1 and the maximum rising rate of LVP and the relationship between the amplitude of dominant peaks/valleys and the corresponding rising rate of LVP were examined by linear, quadratic, cubic, and exponential models. The results showed that the relationships are best fit by nonlinear exponential models.

## Introduction

The mechanisms that determine the intensity of the first heart sound (S1) have been extensively studied during the last century. However, the conclusions of these studies were not well consistent. Sakamoto et al. (1965, 1966) investigated the relationship between the amplitude of S1 and the rising rate of left ventricular pressure (LVP) in 51 dogs using various drugs to invoke wide hemodynamic changes. The results showed that only the peak rising rate of the LVP had a positive linear relationship to the S1 amplitude. This conclusion was widely accepted by subsequent researchers and even applied in the rapid detection of left ventricular systolic dysfunction (Hsieh et al. 2010). Lakier et al. (1972) studied hemodynamic changes in a group of patients with mitral valve diseases and concluded that the more rapid the rise in LVP, the louder the mitral component of S1 becomes. Luisada et al. (1985) conducted experiments in normal young male volunteers who were asked to ride a supine bicycle to modify cardiac dynamics. Their findings suggested that the external phonocardiogram (PCG) was similar to the rate of acceleration of the LVP. Hansen et al. (1989) investigated six anesthetized dogs and concluded that the changes in the amplitude of S1 were closely correlated with the changes in the maximum rising rate of LVP. Stept et al. (1969) examined the effect of changing *P*–*R* interval on the intensity of the mitral component of S1 where the *P*–*R* interval was measured from the beginning of the P wave to the beginning of the QRS-wave. This interval reflects the time the electrical impulse takes to travel from the sinus node through the atrium–ventricle node where it enters the ventricles. The results showed significant increments in the amplitude of the mitral component of S1 occurred at short *P*–*R* intervals; whereas the maximum rising rate of LVP did not change significantly when compared to those at longer *P*–*R* intervals. There was no correlation between the amplitude of the mitral component of S1 and the maximum rising rate at a steady hemodynamic state. Overall, past researches commonly indicated that the amplitude of S1 was positively correlated with the rising rate of LVP. However, the relationship's linearity or nonlinearity was not clear in previous studies.

The purpose of this study was to further investigate this relationship in anesthetized dogs using various doses of epinephrine to invoke a wide range of blood pressures. The study not only examined the relationship between the peak amplitude of S1 and the maximum rising rate but also the relationships between the amplitude of dominant peaks/valleys and the corresponding rising rates.

## Methods

### Experiment settings

This study was approved by the Animal Care Committee of the Chongqing Medical University. Three adult beagle dogs were involved in the experiment with weight between 9 and 11 kg, and with chest wall size between 45 and 50 cm. They were anesthetized with Xylazine (0.2 mL/kg). Additional small doses of Xylazine were given when required, and the dogs were in a supine position during the experiment. A disposable catheter (16G) filled with heparinized solution (500 units/mL) was inserted into the left ventricle via the carotid artery. The catheter was coupled with a pressure transducer (MLT0699; ADInstrument, Bella Vista, NSW, Australia), which had been calibrated under standard atmospheric pressure. Various doses of epinephrine were used to invoke cardiac dynamics, and the channel to inject epinephrine was maintained using an intravenous infusion of 0.9% saline. A microphone transducer (MLT201; ADInstrument) was used to record external PCGs at the apex of the heart. The blood pressure, external PCG, and electrocardiogram (ECG) of lead II were simultaneously sampled at 1 KHz (MP150; BIOPAC, Goleta, CA). The data collection was divided into three stages.

Stage 1: Epinephrine (0.5 μg/kg) was injected. The data collection started 10 sec before the injection and ended when the blood pressure returned to baseline.
Stage 2: Epinephrine (1 μg/kg) was injected. The remaining operations were the same as stage 1
Stage 3: Epinephrine (2 μg/kg) was injected. The remaining operations were the same as stage 1.

The catheter, blood pressure transducer, ECG electrical node, and all electrical lines were fixed during data collection to avoid motion artifacts. The repeated trials for each stage are listed in Table 1. In total, 17 trials were conducted. After the experiments, the dogs were raised until natural death.

**Table 1:** Trial distribution of the experiment

Dog no.	Stage 1 (0.5 μg/kg)	Stage 2 (1 μg/kg)	Stage 3 (2 μg/kg)
1	4	3	2
2	4	×	3
3	1	×	×

### Definitions

**Figure 1:**
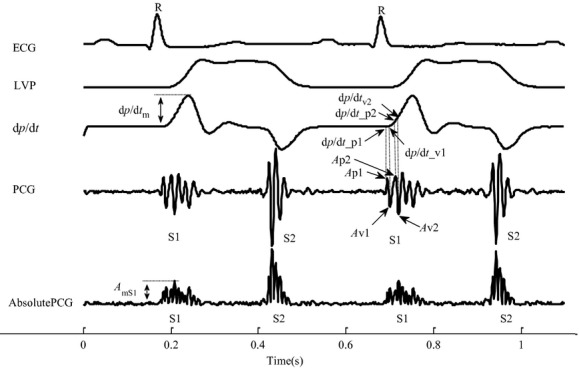
Illustration of the definitions used in this study.

The position of the ECG R-wave was detected using the first differential of the ECG signal and its Hilbert transform (Benitez et al. 2001). The position of the R-wave indicated the start of a cardiac cycle. S1 was segmented as a 200-msec segment starting from the R-wave. The absolute maximum amplitude of S1 was defined as *A*_mS1_. The maximum rising rate of the blood pressure in the left ventricle was defined as d*p*/d*t*_m_. The amplitudes of the first two dominant peaks were defined as *A*_p1_ and *A*_p2_, the amplitudes of the first two dominant valleys were defined as *A*_v1_ and *A*_v2_, and the rising rates of the blood pressure at the first two peaks and the first two valleys were defined as d*p*/d*t*_p1_, d*p*/d*t*_p2_, d*p*/d*t*_v1_, and d*p*/d*t*_v2_. An illustration of these definitions is shown in Figure 1. The objectives of this study were to study five relationships: the relationship between *A*_mS1_ and d*p*/d*t*_m_, the relationship between *A*_p1_ and d*p*/d*t*_p1_, the relationship between *A*_p2_ and d*p*/d*t*_p2_, the relationship between *A*_v1_ and d*p*/d*t*_v1_, and the relationship between *A*_v2_ and d*p*/d*t*_v2_.

### Potential models

Based on conclusions from previous studies and the visual trend of the data in a scatter diagram, four models were examined to fit the relationship between the amplitude and rising rate.

Linear model


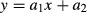
(1)

Quadratic model


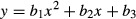
(2)

Cubic model


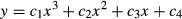
(3)

Exponential model


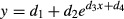
(4)

where *y* was the amplitude; *x* was the rising rate; and *a*_*i*_(*i* = 1, 2), *b*_*i*_(*i* = 1, 2, 3), *c*_*i*_(*i* = 1, 2, 3, 4), and *d*_*i*_(*i* = 1, 2, 3, 4) were the regression coefficients. The regression coefficients were determined using least square criteria.

### Quality index to evaluate fitting

The quality index (*R*^2^) was used to evaluate the goodness of fit of the models (Dougherty et al. 2000) as defined by


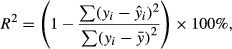
(5)

where *y*_*i*_ was the observed amplitude, 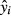 was the amplitude predicted by the model, and 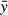 was the mean of *y*_*i*_. The variable *i* was the cycle index. A greater quality index indicated a better fit.

## Results

### Dose response

**Figure 2:**
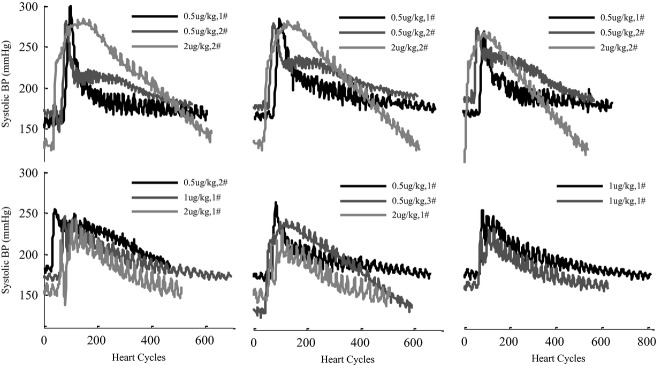
Left ventricular pressure (LVP) responses in the 17 trials.

The dose responses of the 17 trials conducted in this experiment are shown in Figure 2. The LVP increased rapidly after drug injection and decreased gradually over time. The rate of blood pressure decrease varied in the 17 trials. Some decreased quickly from the peak, whereas others decreased slowly. The local fluctuations in LVP were caused by uncontrolled respiration. The relationship between the amplitude of S1 and the cardiac dynamics was investigated in various dose responses.

### Relationship between d*p*/d*t*_m_ and *A*_mS1_

**Figure 3:**
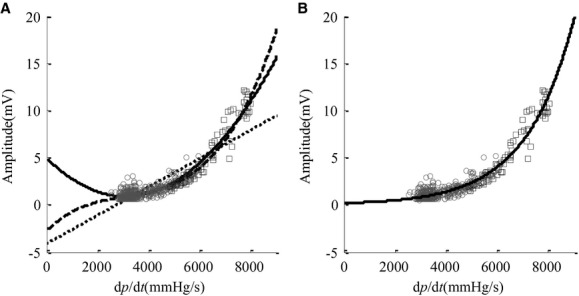
An example of data fitting using the four models. A square indicates a left ventricular pressure (LVP) > 210 mmHg; a circle corresponds to a LVP < 210 mmHg. (A) Data fitting by the quadratic model (solid line), cubic model (dashed line), and linear model (dotted line). (B) Data fitting by the exponential model.

The relationship between d*p*/d*t*_m_ and *A*_mS1_ was investigated in three dogs (17 trials). The relationship was fit by linear, quadratic, and cubic models, as shown in Figure 3A, and an exponential model, as shown in Figure 3B. The fitting quality indexes with a significance confidence level of 0.01 are listed in Table 2. The mean quality indexes in the form of the mean (SD) for the linear, quadratic, cubic, and exponential models were 74.84 (3.62), 79.85 (3.81), 81.00 (3.68), and 82.34 (3.67), respectively. The quality indexes for the linear model were the lowest, and the quality indexes for the exponential model were commonly the highest. Thus, it is logical to conclude that the linear model does not fit the relationship. It was reasonable to adopt the exponential model to fit the relationship based on the quality index analysis.

**Table 2:** Quality indexes of the four models

			*R*^2^			
			
Dose (μg/kg)	Dog no.	Trial	Linear	Quadratic	Cubic	Exponential
0.5	1	1	77.62	82.31	82.32	82.19
		2	76.44	83.80	84.28	84.35
		3	78.27	81.83	82.88	82.95
		4	78.17	79.02	79.35	83.84
	2	1	70.37	74.63	75.66	76.37
		2	74.65	78.74	79.63	79.49
		3	73.79	78.03	79.46	78.88
		4	68.39	80.68	82.80	83.18
	3	1	77.31	78.22	78.51	81.36
1	1	1	70.13	76.21	77.32	79.21
		2	77.73	84.51	86.08	86.73
		3	77.36	78.82	79.37	79.82
2	1	1	67.10	72.73	76.69	79.72
		2	75.12	75.25	75.95	78.61
	2	1	75.71	82.97	83.86	86.62
		2	76.49	83.37	85.24	86.49
		3	77.54	86.27	87.63	90.03
Max			78.27	86.27	87.63	90.03
Min			67.10	72.73	75.66	76.37
Mean			74.84	79.85	81.00	82.34
SD			3.62	3.81	3.68	3.67

Another principle used to select a fitting model is the extension of the model. As shown in Figure 3A, the quadratic and cubic models fit the data well and had high-quality indexes. However, the quadratic and cubic models had wrong extension out the data. The quadratic model rose rapidly, and the cubic model decreased quickly as d*p*/d*t*_m_ approached zero. The wrong extensions of the two models were obviously inconsistent with the physical meaning of the data. The extension of the exponential model, however, had right extension out the data. The amplitude decreased, but remained positive as d*p*/d*t*_m_ approached zero. Student's *t*-test showed that the exponential model fit the data in the 17 trials.

Considering the findings mentioned above, the relationship between *A*_mS1_ and d*p*/d*t*_m_ was fitted by the exponential model


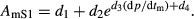
(6)

The regression coefficients for the 17 trials are listed in Table 3. Based on the previous studies, 210 mmHg is selected as a threshold of high LVP. The higher and the lower LVP were indicated by squares and circles in Figure 3A and B, respectively. It can be found that the circles may present a positive linear relation for low LVP.

**Table 3:** Regression coefficients of the exponential model for *A*_mS1_ and d*p*/d*t*_m_

			Regression coefficients			
			
Dose (μg/kg)	Dog no.	Trial	*d*_1_*	*d*_2_*	*d*_3_* × 10^−3^	*d*_4_*
0.5	1	1	−0.03	0.22	0.56	−0.16
		2	0.32	0.13	0.64	−0.52
		3	−0.28	0.21	0.56	−0.12
		4	−2.43	0.50	0.32	0.78
	2	1	0.95	0.02	1.37	−1.63
		2	0.58	0.10	0.67	−0.72
		3	0.48	0.14	0.58	−0.38
		4	0.72	0.09	0.77	−0.86
	3	1	0.91	0.02	0.92	−2.20
1	1	1	0.99	0.09	0.80	−0.88
		2	1.05	0.09	0.85	−0.94
		3	−0.15	0.32	0.49	0.22
2	1	1	2.45	0.06	0.10	−1.28
		2	−2.80	0.76	0.24	1.17
	2	1	1.67	0.11	0.42	−0.46
		2	1.64	0.08	0.40	−0.02
		3	1.61	0.08	0.45	−0.42

* Regression coefficients of equation (8).

### Relationships between d*p*/d*t*_p1_, d*p*/d*t*_p2_ and *A*_p1_, *A*_p2_

**Figure 4:**
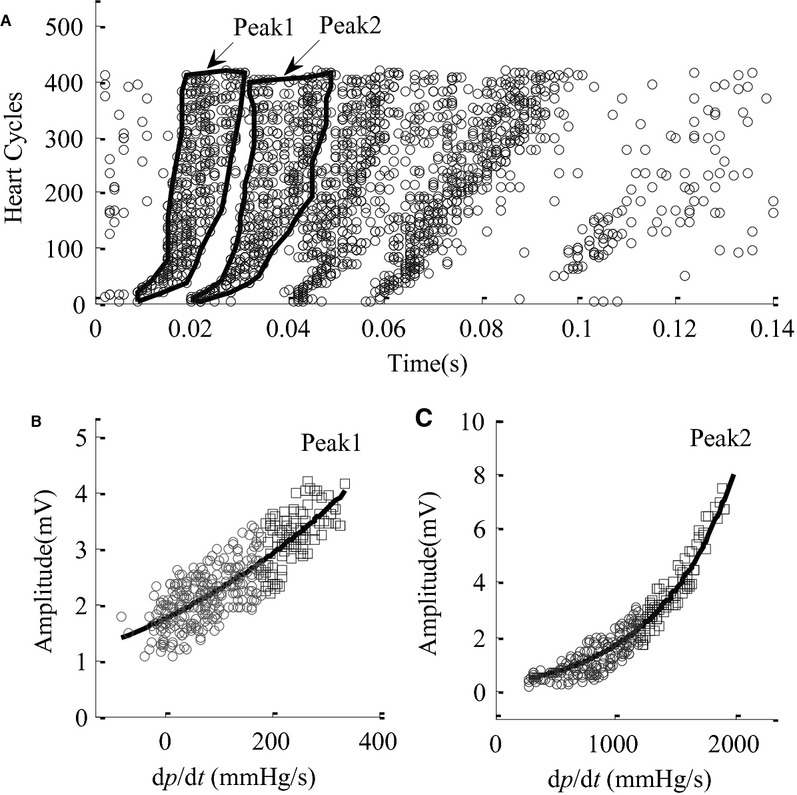
An example of model fitting for the peaks. A square indicates a left ventricular pressure (LVP) > 210 mmHg; a circle corresponds to a LVP < 210 mmHg. (A) Scatter plot of the peaks with respect to cardiac cycles. The two polygons indicate the first two dominant peaks. (B, C) Scatter diagrams of the peak amplitudes with respect to the corresponding pressure rising rates. The solid lines are the fitted curves.

All the S1s of a record with respect to the R-wave were segmented cycle by cycle. The peak S1s are indicated by circles in Figure 4A; several clusters were found. The two clusters enclosed by the polygons in Figure 4A correspond to the first two dominant peaks, Peak 1 and Peak 2. Scatter plots showing the relationships between the amplitudes of the two peaks and the corresponding pressure rising rates are given in Figure 4B and C. Based on the above analysis, the exponential model was used to fit the relationships between d*p*/d*t*_p1_, d*p*/d*t*_p2_ and *A*_p1_, *A*_p2_. The solid lines in Figure 4B and C show the regression curves for the two peaks.

The relationships between d*p*/d*t*_p1_, d*p*/d*t*_p2_ and *A*_p1_, *A*_p2_ were fitted by the exponential model


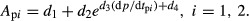
(7)

The regression coefficients of the two peaks with a significance confidence level of 0.01 are listed in Table 4.

**Table 4:** Regression coefficients of the exponential model for the first two dominant peaks

			Regression coefficients							
			
			Peak 1				Peak 2			
				
Dose (μg/kg)	Dog no.	Trial	*d*_1_*	*d*_2_*	*d*_3_* × 10^−3^	*d*_4_*	*d*_1_*	*d*_2_*	*d*_3_* × 10^−3^	*d*_4_*
0.5	1	1	0.16	0.32	10.55	0.06	0.37	0.03	4.70	−0.84
		2	0.37	0.12	2.98	−0.36	−0.34	0.27	0.66	−0.13
		3	0.20	0.13	2.65	−0.13	0.11	0.01	1.33	1.33
		4	0.26	0.15	2.52	−0.46	−0.25	0.22	0.87	−0.28
	2	1	0.95	0.17	11.58	−0.57	0.13	0.20	1.55	−0.19
		2	0.72	0.15	13.91	−0.75	0.23	0.15	1.63	−0.58
		3	0.58	0.25	5.31	−0.30	0.27	0.12	1.63	−0.13
		4	0.70	0.18	9.33	−0.57	0.03	0.30	1.03	−0.03
	3	1	−0.04	0.24	8.83	−0.25	−0.16	0.20	1.26	−0.44
1	1	1	0.29	0.19	2.37	−0.43	−27.02	2.77	0.03	2.26
		2	−0.06	0.32	1.48	0.11	−16.33	2.21	0.05	1.97
		3	−27.94	3.16	0.28	2.22	0.20	0.23	1.86	−0.03
2	1	1	−0.57	0.91	2.02	0.93	0.45	0.21	1.91	−0.13
		2	−13.57	2.31	0.39	1.88	−0.68	0.53	1.13	0.43
	2	1	−13.25	2.21	0.19	1.86	0.60	0.35	0.48	0.26
		2	−0.83	1.40	0.34	1.40	0.86	0.28	0.54	0.05
		3	0.49	0.38	3.43	0.10	0.76	0.28	0.57	0.03
Quality index (mean±SD)			77.97 ± 3.92				80.55 ± 6.41			

* Regression coefficients of equation (7).

### Relationships between d*p*/d*t*_v1_, d*p*/d*t*_v2_ and *A*_v1_, *A*_v2_

**Figure 5:**
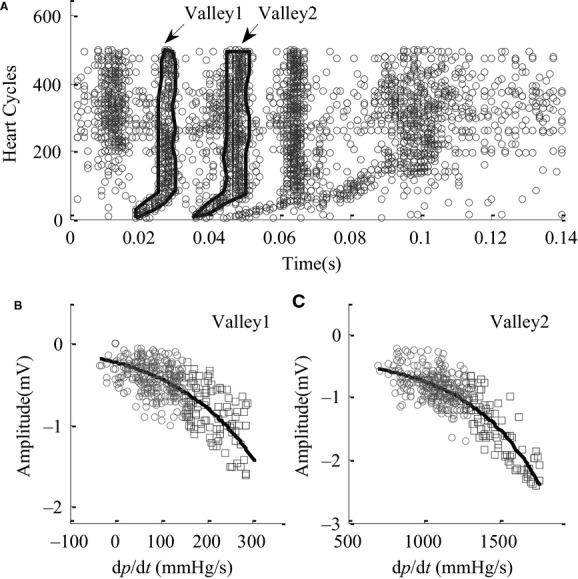
An example of model fitting for the valleys. (A) Scatter plot of the valleys with respect to cardiac cycles. The two polygons indicate the first two dominant valleys. (B, C) Scatter diagrams of the valley amplitudes with respect to the corresponding pressure rising rates. The solid lines are the fitted curves.

Similar to the analysis performed in Relationships between d*p*/d*t*_p1_, d*p*/d*t*_p2_ and *A*_p1_, *A*_p2_, the valleys of all the recorded S1s are plotted cycle by cycle in Figure 5A. The two clusters enclosed by the polygons correspond to Valley 1 and Valley 2. The scatter plots showing the relationships between the amplitudes of the two valleys, and the corresponding pressure rising rates are given in Figure 5B and C. The solid lines show the fitted curves based on the exponential model.

The relationships between d*p*/d*t*_v1_, d*p*/d*t*_v2_ and *A*_v1_, *A*_v2_ were fitted using the exponential model


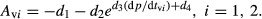
(8)

Table 5 shows the regression coefficients with a significance confidence level of 0.01.

**Table 5:** Regression coefficients of the exponential model for the first two dominant valleys

			Regression coefficients							
			
			Valley 1				Valley 2			
				
Dose (μg/kg)	Dog no.	Trial	*d*_1_*	*d*_2_*	*d*_3_* × 10^−3^	*d*_4_*	*d*_1_*	*d*_2_*	*d*_3_* × 10^−3^	*d*_4_*
0.5	1	1	−0.01	0.27	5.98	−0.12	0.14	0.13	1.64	−0.11
		2	−0.28	0.41	4.97	0.26	−2.32	0.83	0.40	0.88
		3	−21.11	2.65	0.14	2.09	−0.52	0.41	0.89	0.23
		4	−7.74	1.64	0.46	1.59	−0.57	0.41	0.88	0.24
	2	1	0.22	0.28	9.89	−0.03	−18.83	2.52	0.07	2.03
		2	0.26	0.12	2.41	−0.98	0.25	0.22	5.37	−0.36
		3	0.33	0.02	31.42	−2.45	−24.86	2.91	0.09	2.16
		4	0.35	0.05	24.04	−1.64	−20.69	2.70	0.10	2.06
	3	1	−1.80	0.79	0.80	0.87	−0.24	0.26	0.65	−0.11
1	1	1	−27.35	3.04	0.12	2.22	−44.69	3.76	0.03	2.47
		2	−0.45	0.54	6.12	0.50	−31.33	3.30	0.08	2.28
		3	−18.43	2.59	0.13	2.01	−49.78	3.79	0.04	2.56
2	1	1	−0.15	0.57	3.95	0.51	−5.40	1.53	0.29	1.47
		2	−2.06	0.90	3.07	1.01	−8.77	1.87	0.21	1.67
	2	1	0.41	0.14	12.97	−0.63	−32.37	3.38	0.03	2.30
		2	0.48	0.08	17.34	−1.22	−42.12	3.81	0.03	2.43
		3	−5.65	1.44	0.16	1.46	−27.49	2.98	0.05	2.25
Quality index (mean±SD)			78.81 ± 4.60				81.52 ± 5.44			

* Regression coefficients of equation (10).

## Discussion

Previous studies commonly concluded that the amplitude of S1 was linearly related to the blood pressure rising rate. However, the results in this study showed with high confidence that the relationship is nonlinear exponential. Analyzing the reasons for this inconsistency is important, and the reasons are as follows.

1. Most of the previous studies were conducted before the 1970s; thus, the signal recording systems used were analog. The first derivative of LVP was calculated using an analog circuit, and the S1 amplitudes and rising rates were measured using a ruler on the trace lines printed on a piece of paper with a paper feed speed of 100 or 200 mm/sec. Measurement and timing errors were unavoidable. Conversely, the signals in this study were sampled using a modern advanced digital system. The amplitudes and timing were accurately obtained by A/D conversion; therefore, the measurement errors in this study are negligible.
2. The frequency band of the S1s being analyzed have an impact on amplitude measurement. For example, Sakamoto et al. (1965) filtered the heart sound signal of dogs using high-pass filters with cutoff frequencies of 100 and 200, or 50 Hz occasionally. In the experiments by Luisada et al. (1958), the heart sound signal of dogs was between 60 and 100 Hz, or 60 and 250 Hz occasionally. However, the power spectral analysis in this study showed that the dominant energy of dog heart sounds is between 30 and 200 Hz. Therefore, there was a mismatch between the heart sound frequency band and the filter cutoffs in the previous studies. The amplitude of the heart sounds may have been reduced to some degree due to this mismatch. The frequency band of the heart sound transducer used in this study, however, was 0.5–1000 Hz at 3 dB, and the signal was linearly amplified by an amplifier with a frequency band between 5 and 1000 Hz. Thus, the heart sounds remained intact to a high degree in this study.
3. The ranges of the LVP and rising rate in this study were commonly greater than those of previous studies. The ranges of the LVP and LVP in the study by Sakamoto et al. (1965), which were calculated by the authors from their illustrations, were from 70 to 250 mmHg and from 500 to 3000 mmHg/sec, respectively. A visual check by the authors on the scatter plot provided by Sakamoto et al. (1965) showed that the relationship was not quite linear at larger rising rate invoked by epinephrine, although the linear relationship was most significant at lower rising rates invoked by other drugs such as norepinephrine and methoxamine. However, the slight nonlinearity was neglected by Sakamoto. In Luisada's study, the range of volunteers’ LVP ranged from 110 to 210 mmHg (Luisada et al. 1985). In the experiments by Hansen et al. (1989), the rising rate of the left ventricle varied between 750 and 5000 mmHg/sec. In this study, various doses of epinephrine were used to invoke wider hemodynamic changes: the LVP varied from 130 to 290 mmHg, the maximum rising rate varied from 2000 to 8000 mmHg/sec, and the rising rate of corresponding peaks/valleys varied from 0 to 3000 mmHg/sec. Therefore, the exponential relationships found in this study were determined using a larger range of data. These relationships could potentially reduce to linearity if the analysis was conducted within a smaller data range, as indicated by the gray circles in Figures 5. The authors believe this is the main reason for the inconsistency.

## Conclusions

The relationship between the amplitude of S1 and the rising rate of LVP was investigated in anesthetized dogs using various doses of epinephrine. Linear, quadratic, cubic, and exponential models were examined to fit the relationship. The results showed that the relationship between the maximum amplitude of S1 and the maximum rising rate of LVP, and the relationships between the amplitude of the dominant peaks/valleys and the corresponding LVP rising rates were all exponential and not linear. The main cause of the inconsistency with previous studies was the large range of data that was used in this study.
